# CT of the paraumbilical and ensiform veins in patients with superior vena cava or left brachiocephalic vein obstruction

**DOI:** 10.1371/journal.pone.0196093

**Published:** 2018-04-26

**Authors:** Joseph Casullo, Han Zeng, Geneviève Belley, Giovanni Artho

**Affiliations:** Department of Diagnostic Radiology, Montreal General Hospital, McGill University Health Center, Montreal, Quebec, Canada; Seoul National University Hospital, REPUBLIC OF KOREA

## Abstract

The purpose of this study was to elaborate on the anastomoses between the paraumbilical and systemic veins, particularly the ensiform veins. The connections with the ensiform veins have received little attention in the anatomical and radiological literature, and remain incompletely described. Too small to be reliably traced in normal CT scans, the paraumbilical veins can dilate in response to increased blood flow from systemic veins in superior vena cava obstruction (SVCO), allowing a study of their arrangement and connections. Collateral paraumbilical veins were therefore analyzed retrospectively in 28 patients with SVCO using CT. We observed inferior and superior groups of collateral vessels in 23/28 (82%) and 17/28 (61%) patients, respectively. Inferior veins ascended towards the liver and drained into portal veins (19/28, 68%) or the umbilical vein (8/28, 29%); superior veins descended and drained into portal veins. The inferior veins (N = 27) could be traced to ensiform veins in almost all of the cases (26/27, 96%), and a little over half (14/27, 52%) were also traceable to subcutaneous and deep epigastric veins. They were opacified by ensiform (25/27, 93%), deep epigastric (4/27, 15%) and subcutaneous (4/27, 15%) veins. The superior veins (N = 17) were supplied by diaphragmatic (13/17, 76%) and ensiform veins (4/17, 24%); the diaphragmatic veins were branches of collateral internal thoracic, left pericardiacophrenic and anterior mediastinal veins. Collateral ensiform veins were observed in 22 patients and anastomosed with internal thoracic (19/22, 86%), superior epigastric (9/22, 41%), diaphragmatic (4/22, 18%), subcutaneous (3/22, 14%) and anterior mediastinal veins (1/22, 5%). These observations show that the paraumbilical veins communicate with ensiform, deep epigastric, subcutaneous and diaphragmatic veins, joining the liver to the properitoneal fat pad, anterior trunk, diaphragm and mediastinum. In SVCO, the most common sources of collateral flow to the paraumbilical veins are the ensiform and diaphragmatic branches of the internal thoracic veins.

## Introduction

The paraumbilical veins are small accessory portal veins confined between the layers of the falciform ligament. They join the caval and portal systems by communicating with systemic veins at one end and the portal circulation at the other. Three groups have been described: the veins of Burow and the superior and inferior veins of Sappey [1].

**Fig 1:**
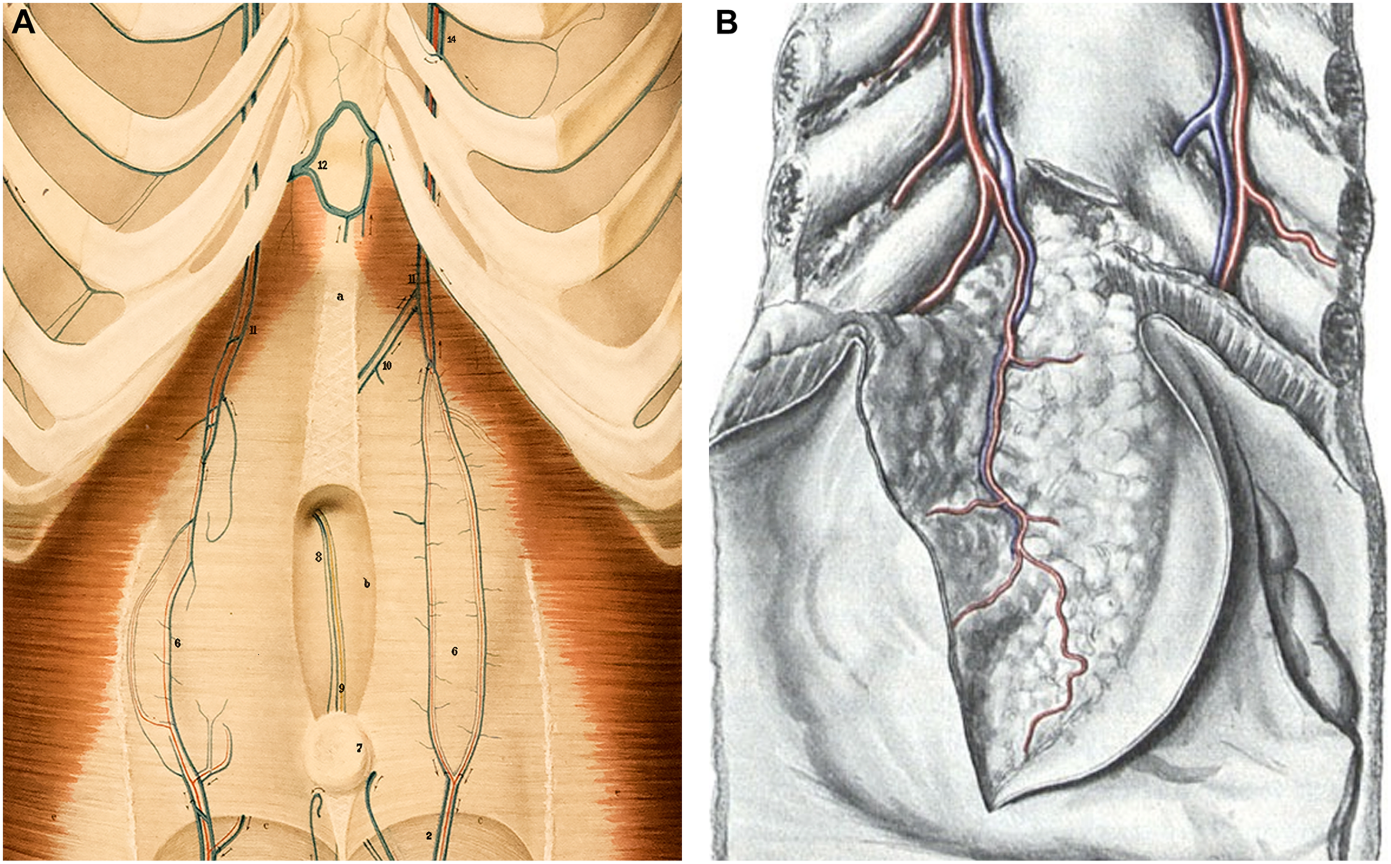
Anatomical depictions of the paraumbilical and ensiform veins. (A) Detail of Braune’s 1884 color lithograph of the veins of the anterior trunk, Tafel (plate) IV. The muscles were partially removed to uncover the internal thoracic (14) and epigastric veins. The superior (11) and inferior epigastric (2) veins, and their deep tributaries (6) lie superficial to the posterior layer of the rectus sheath. Also exposed are the subaponeurotic obliterated umbilical vein (9) and adjacent inferior Sappey veins (8) ascending from the umbilicus (7) before disappearing in the falciform ligament. A pair of venae paraumbilicalis xyphoidae (10) enter the left superior epigastric vein after piercing the rectus sheath next to the linea alba (a). There are also two unnamed vessels that enter a transverse vein (12) bridging the internal thoracic veins at the xiphoid process. These vessels are likely ensiform veins ascended from the properitoneal fat pad deep to the rectus sheath except at the very top. (Courtesy US National Library of Medicine) (B) Illustration of the properitoneal fat pad and the ensiform branches of the left internal thoracic vessels in a specimen, posterior view. The parietal peritoneum was opened and turned aside, and the xiphoid process was resected. (From Nordenson et al. (1930), by permission of Springer).

Burow [2], Sappey [3, 4], Braune [5], Baumgarten [6], Butler [7], Martin & Tudor [1], and Ibukuro et al. [8] reported direct anastomoses between the paraumbilical veins and the internal thoracic-epigastric trunks, diaphragmatic veins and subcutaneous veins in their post-mortem anatomical studies. Braune also observed anastomoses between Sappey’s inferior veins and tributaries of the left superior epigastric vein that ascended deep to the rectus sheath. These vessels were named the venae paraumbilicalis xyphoidea by Braune (Fig 1A) and bear a resemblance to the ensiform (latin equivalent for xiphoid) veins, which Nordenson et al. [9] and Merklin [10] later described in their investigations on the properitoneal fat pad (Fig 1B). The ensiform veins accompany the ensiform artery [11] as they traverse the properitoneal fat pad and are tributaries of the superior epigastric and internal thoracic vessels. The connections between the paraumbilical and ensiform veins have received little attention in the literature since Braune first discovered them and remain incompletely described.

The purpose of this study was to elaborate on the relationship between the paraumbilical veins and the systemic vessels, particularly the ensiform veins. In the CT scans of normal subjects, the paraumbilical veins are not easily traceable because of their size. However, they can expand in response to collateral blood flow from the systemic veins in superior vena cava (SVC) obstruction [12, 13, 14], lending themselves to a study of their anastomoses. We therefore analyzed the arrangement and connections of collateral paraumbilical veins in the CT scans of patients with chronic superior venous obstruction. Observations from the previous anatomical studies are compared, and clinical considerations are discussed.

## Material and methods

We observed the paraumbilical veins in 28 patients recruited from the authors’ database of chronic SVC obstruction. The patient’s CT scans, performed between March 2006 and January 2014, were analyzed retrospectively. Indications for the examinations varied; Table 1 lists the different CT protocols used. The Mcgill University Hospital Center Research Ethics Board approved this study and waived the requirement for informed patient consent.

**Table 1:** Clinical features of 28 patients with superior vena cava (SVC) or left brachiocephalic vein (LBCV) obstruction and collateral paraumbilical veins.

*Clinical Feature*	*Value*
***Sex*, *no*. *of patients***	
Female	17
Male	11
***Age*, *y***	
Mean	60
Range	34–83
***CT protocol used*, *no*. *(%) of patients***	
CT chest, routine contrast-enhanced	13 (46)
CT chest & abdomen, routine contrast-enhanced	4 (15)
CT chest, venogram	4 (15)
CT chest & abdomen, venogram	2 (7)
CT pulmonary angiogram	2 (7)
CT liver, multiphasic	2 (7)
CT abdomen, routine contrast-enhanced	1 (4)
***Cause of SVC or LBCV obstruction*, *no*. *(%) of patients***	
Malignancy (paratracheal mass)	14 (50)
Catheter-related thrombosis	9 (32)
Post-surgical and radiation injury	2 (7)
Chronic fibrosing mediastinitis	2 (7)
Anatomic compression of LBCV	1 (4)
***Site of venous obstruction*, *no*. *(%) of patient***	
SVC^a^ with retrograde azygos return	20 (71)
SVC^a^ with antegrade azygos return	7 (25)
Lower LBCV	1 (4)
***Side of injection*, *no*. *(%) of patients***	
Left	13 (46)
Right	9 (33)
Bilateral	6 (21)

^**a**^With variable occlusion of the azygos and brachiocephalic veins

Patients were between 34 and 83 years old with an average of 60 years. Table 1 shows the causes and sites of venous obstruction. In the majority of the cases, the obstruction was caused by a malignant right paratracheal mass (50%) or venous thrombosis related to indwelling or remote catheters (32%). The lower third of the left brachiocephalic vein was anatomically compressed between the innominate artery and the manubrium [15] in one patient (4%) with lymphoma in remission. Of the 14 patients with a malignant right paratracheal mass, 11 had lung malignancy, two had lymphoma and one had metastatic adenopathy from breast carcinoma. Of the nine patients with catheter-related thrombosis, six had underlying malignancy and three had long-term dialysis catheters.

Studies were performed on 64-detector CT scanners (LightSpeed VCT, LightSpeed Discovery CT750 HD; GE Healthcare, Milwaukee, Wisc., USA). The following parameters were used: 120 kVp, automatic current tube modulation with a maximum of 550–600 mA, noise index of 21 or 27.6, tube rotation time of 0.7 s and pitch of 0.984:1. Patients received non-ionic contrast material (iohexol [Omnipaque 300 mg/mL, GE Healthcare] or iodixanol [Visipaque 320 mg/mL, GE Healthcare]) through upper extremity veins; the side of injection is recorded in Table 1.

Routine CT scans of the chest were obtained with 1.25 × 1.25 mm collimation and no overlap. CT-angiograms, CT-venograms and CT studies of the abdomen, which included the pelvis, were obtained with 1.25 mm collimation at 0.9 mm intervals.

For the 18 routine CT scans of the chest and/or abdomen, patients received 80–125 mL of contrast material at a rate of 2 mL/s. Depending on the study, the scanning delay was between 50 and 70 seconds, or was determined using bolus-tracking software (SmartPrep; GE Healthcare).

For the six CT venographic studies, patients received 50 mL of contrast material in each arm simultaneously at a rate of 3 mL/s, and the scanning delay was 25 seconds.

Pulmonary CT angiography was performed in two patients; a bolus of 55 mL of contrast material followed by a 35 mL saline flush was injected at 4.5 mL/s. The scanning delay was predetermined by a test bolus (20 mL) using the pulmonary trunk as the region of interest.

Two patients had a multiphasic CT scan of the liver. Following a non-contrast-enhanced scan of the liver, the liver was rescanned after the patients received 80–100 mL of contrast material at a rate of 3 ml/s. The scanning delay was determined using bolus-tracking software (SmartPrep; GE Healthcare). Delayed phase images of the abdomen, and then the liver, were obtained at 70 seconds and 5 minutes, respectively.

We performed volume rendering (maximum intensity projection) at the workstation using embedded software.

## Background anatomy

Before describing our results, the anatomy of the paraumbilical and ensiform veins will be briefly reviewed. The information is based on post-mortem studies of normal subjects or of cases of chronic liver disease.

Sappey’s superior veins drain the median diaphragm and communicate with the diaphragmatic and internal thoracic veins [1, 3, 4, 8]. They traverse the upper part of the falciform ligament to terminate at the convex surface of the liver where they anastomose with peripheral branches of the left portal vein (LPV). These veins form an array of arches between the layers of the falciform ligament and differ in mural structure from the other paraumbilical veins [1].

Sappey’s inferior veins traverse the lower part of the falciform ligament as they ascend to the liver. Sappey [3, 4], and Martin & Tudor [1] described a main channel that drains into the LPV, the quadrate lobe directly or uncommonly into the umbilical vein [1, 5], and receives multiple tributaries that communicate with the inferior epigastric and subcutaneous veins. Braune [5] identified a connection between the main channel and the left superior epigastric vein through the venae paraumbilicalis xyphoidea, which ascend deep to the rectus sheath and resemble the ensiform veins [9, 10, 16]. Sappey [3] also described a separate group of smaller inferior veins that follow the umbilical vein and end directly in the liver, the LPV, or the umbilical vein [17].

Burow’s veins ascend from the inferior epigastric veins and adhere to the umbilical vein. These veins drain into the umbilical vein; none enter the liver directly [1, 2, 7]. (In the adult, the lumen of the umbilical vein persists over a distance of 14–16 cm from the LPV [18] and delivers blood from systemic and paraumbilical tributaries to the portal system. Below this patent part, the lumen is obliterated by subendothelial proliferation of connective tissue and fragmentation of the vein wall into fibrous strands that attach to the rectus sheath at the umbilicus [1].)

The ensiform veins, tributaries of the superior epigastric and internal thoracic veins, drain the properitoneal fat pad, which rests between the parietal peritoneum and the rectus sheath from the umbilicus to the diaphragm [9, 10, 16]. When they drain into the internal thoracic veins they cross over the xiphoid process [10].

## Results

In the majority of the cases, the paraumbilical veins were brightly opacified, measuring 0.5–4 mm in diameter. Inferior and superior veins were identified. One or two inferior veins had become dilated in 23 patients (23/28, 82%), and a dilated superior vein was found in 17 patients (17/28, 61%). Twelve patients (12/28, 43%) had both inferior and superior veins. The inferior veins communicated directly with portal veins (19/28, 68%) or drained into the umbilical vein (8/28, 29%), and the superior veins communicated with portal veins.

### Inferior veins communicating directly with portal veins

**Fig 2:**
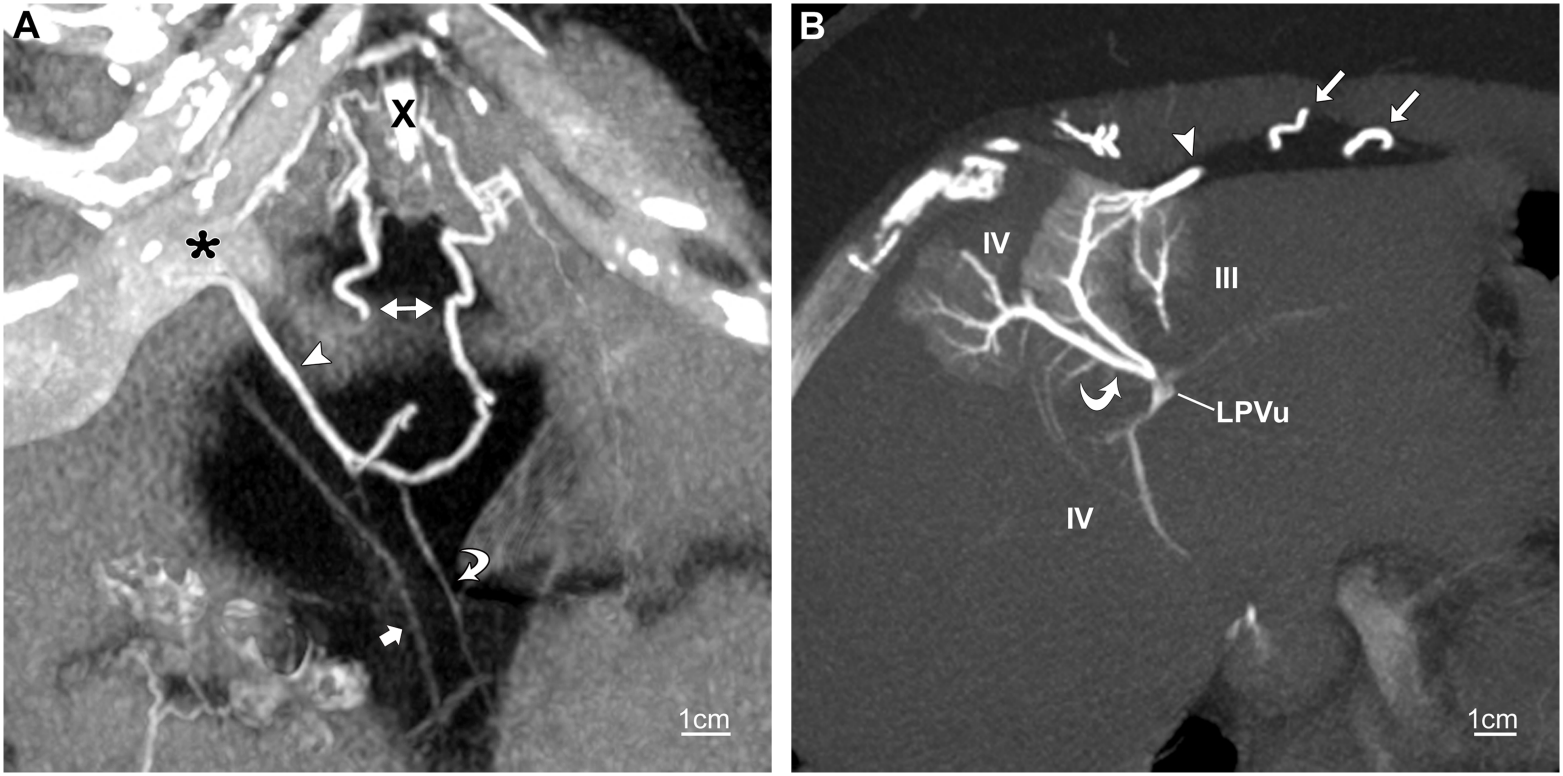
Inferior paraumbilical vein. CT venogram of chest in a 56-year-old woman with SVC obstruction. (A) Slightly oblique 7.5-mm coronal maximum-intensity-projection (MIP) shows an inferior paraumbilical vein (arrowhead) terminating at the umbilical notch of the liver (asterisk). It is predominantly opacified by two tributaries continuous with ensiform vessels (right-left arrow). A third tributary (curved arrow) joins a subcutaneous vein (not shown). The apparent discontinuity in the right ensiform vessel is due to tortuosity. Also shown is the umbilical vein (straight arrow). X, xiphoid process. (B) Oblique transverse 7-mm MIP demonstrates the inferior vein (arrowhead) joining dilated terminal portal vessels in segments IV and III at the margin of the liver. The terminal vessels in segment IV drain into an expanded second- or third-order portal branch that empties into the distal left portal vein (LPVu) resulting in antegrade opacification of an adjacent second- or third-order portal branch (curved arrow) in segment IV and of portal branches in segments III and IV posteriorly. The collateral ensiform veins (straight arrows) are shown in the properitoneal fat pad.

**Fig 3:**
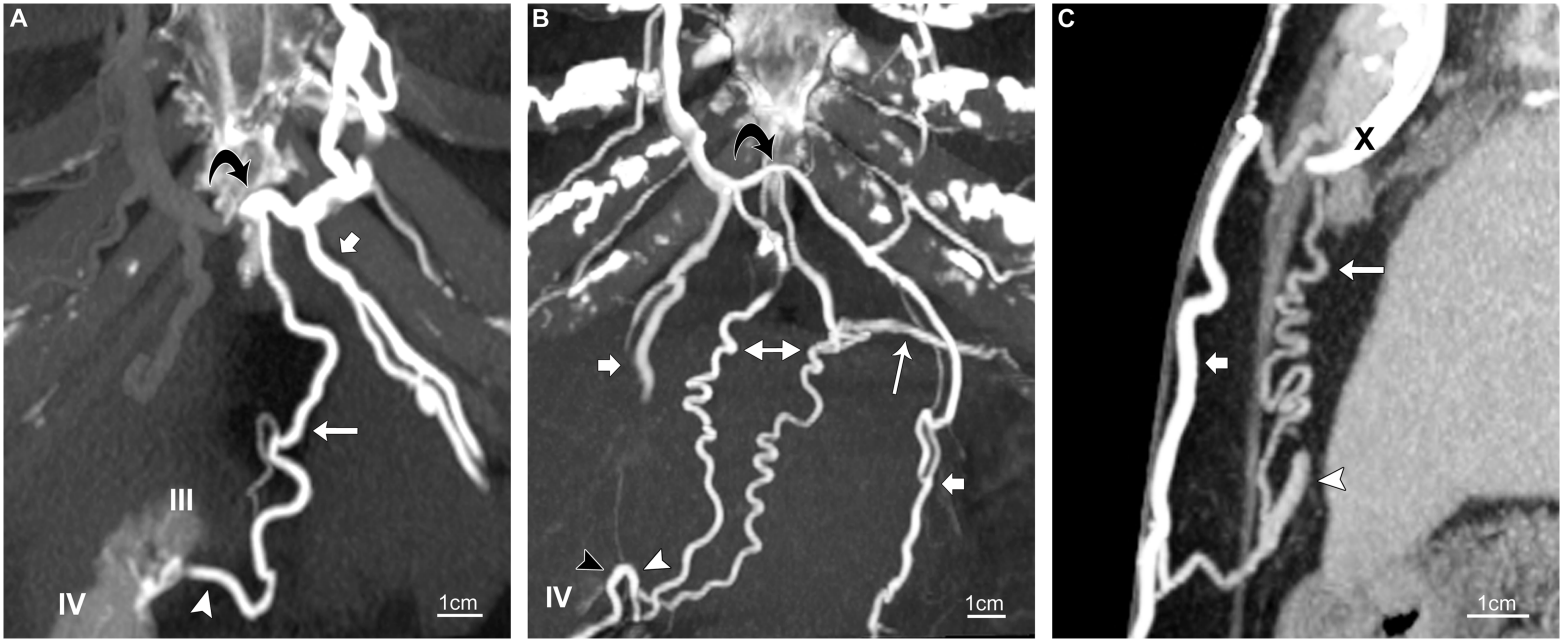
Inferior paraumbilical veins. Routine contrast-enhanced CT scans of chest in three patients with SVC obstruction. (A) 58-year-old woman. Coronal 10-mm maximum-intensity-projection (MIP) shows an inferior paraumbilical vein (arrowhead) terminating at the umbilical notch. It joins subcutaneous veins (not shown), but its only source of collateral flow is an ensiform vein (long arrow) descended from a transverse vessel (curved arrow) between the internal thoracic veins at the xiphoid process. The inferior vein communicates with segments IV and III. The left superior epigastric vein (short arrow) is also shown. (B) 70-year-old woman. Coronal 20-mm MIP shows the confluence of two inferior paraumbilical veins (arrowheads) at the free edge of segment IV. One vessel (black arrowhead) communicates with deep epigastric veins (not shown); the other (white arrowhead) receives two tributaries continuous with ensiform vessels (right-left arrow). The ensiform veins are opacified by the right internal thoracic vein via a transverse branch (curved arrow) across the xiphoid process, and also communicate with left diaphragmatic veins (long arrow). Short arrows point to superior epigastric veins. (C) 76-year-old woman. Sagittal 10-mm MIP shows an inferior paraumbilical vein (arrowhead) destined for segment III. It communicates with a collateral subcutaneous vein (short arrow) across the linea alba and with an ensiform vessel (long arrow). The subcutaneous and ensiform veins both join a transverse vein (not shown) bridging the internal thoracic veins at the xiphoid process (X).

A main vessel ascended to the liver to terminate at its free margin in 19 patients (Figs 2 and 3; Parts A—E in S1 Fig). In 10 (53%) of these patients, the main vessel could be traced to deep epigastric or subcutaneous veins at or a short distance above the umbilicus. It also received one to three tributaries that were continuous with single or paired ensiform veins running through the properitoneal fat pad. In the other nine (47%) cases, the main vessel joined only ensiform veins; a direct connection with deep epigastric or subcutaneous veins was not identified. The main vessel was opacified by collateral ensiform veins in almost all of the cases (18/19, 95%), and in four of them, it also received collateral flow from deep epigastric (2/19, 11%) or subcutaneous veins (2/19, 11%). In one case (1/19), only subcutaneous veins supplied the main vessel (Table 2).

**Table 2:** Sources of collateral flow to the paraumbilical veins.

*Sources*	*No*. *(%) of Patients*
***Inferior veins communicating directly with portal veins (n = 19)***	
Ensiform veins	18 (95)
Subcutaneous veins	3 (19)
Deep epigastric veins	2 (15)
***Inferior veins communicating with umbilical vein (n = 8)***	
Ensiform veins	7 (88)
Deep epigastric veins	2 (25)
Subcutaneous veins	1 (13)
***Superior group (n = 17)***	
Left internal thoracic veins^a^	6 (35)
Right internal thoracic veins^a^	5 (29)
Ensiform veins	4 (24)
Left pericardiacophrenic veins^a^	2 (12)
Left and right internal thoracic veins^a^	1 (6)
Mediastinal veins^a^	1 (6)

^a^via diaphragmatic branches

Focal contrast-enhancement of the liver occurred in 18 patients (18/19, 95%). Anastomoses between the main vessel and dilated peripheral branches of the umbilical portion of the left portal vein (LPVu) were seen in twelve cases (12/18, 67%) (Figs 2B and 3A). In one case, the main vessel was insufficiently opacified to produce liver enhancement. Table 3 shows the distribution of the opacified portal segments, which were determined by tracing the terminal divisions of second order portal branches of the LPVu to the opacified areas [19, 20]. In four cases, contrast medium flowed from peripheral portal veins in segment IV into the distal end of the LPVu (Fig 2B) where it was redistributed to other second order branches of segment IV and to segments III and II.

**Table 3:** Distribution of the portal segments opacified by collateral paraumbilical veins.

*Portal Segment*	*No*. *(%) of Patients*
***Inferior veins communicating directly with portal veins (n = 18)***	
Segment IV	16 (89)
Segment III	**7** (39)
Segment II	1 (5)
***Inferior veins communicating with umbilical vein (n = 6)***	
Segment IV	6 (100)
Segment III	5 (83)
***Superior veins (n = 17)***	
Segment IV	14 (82)
Segment III	1 (6)
Indeterminate	2 (12)

### Inferior veins entering the umbilical vein

**Fig 4:**
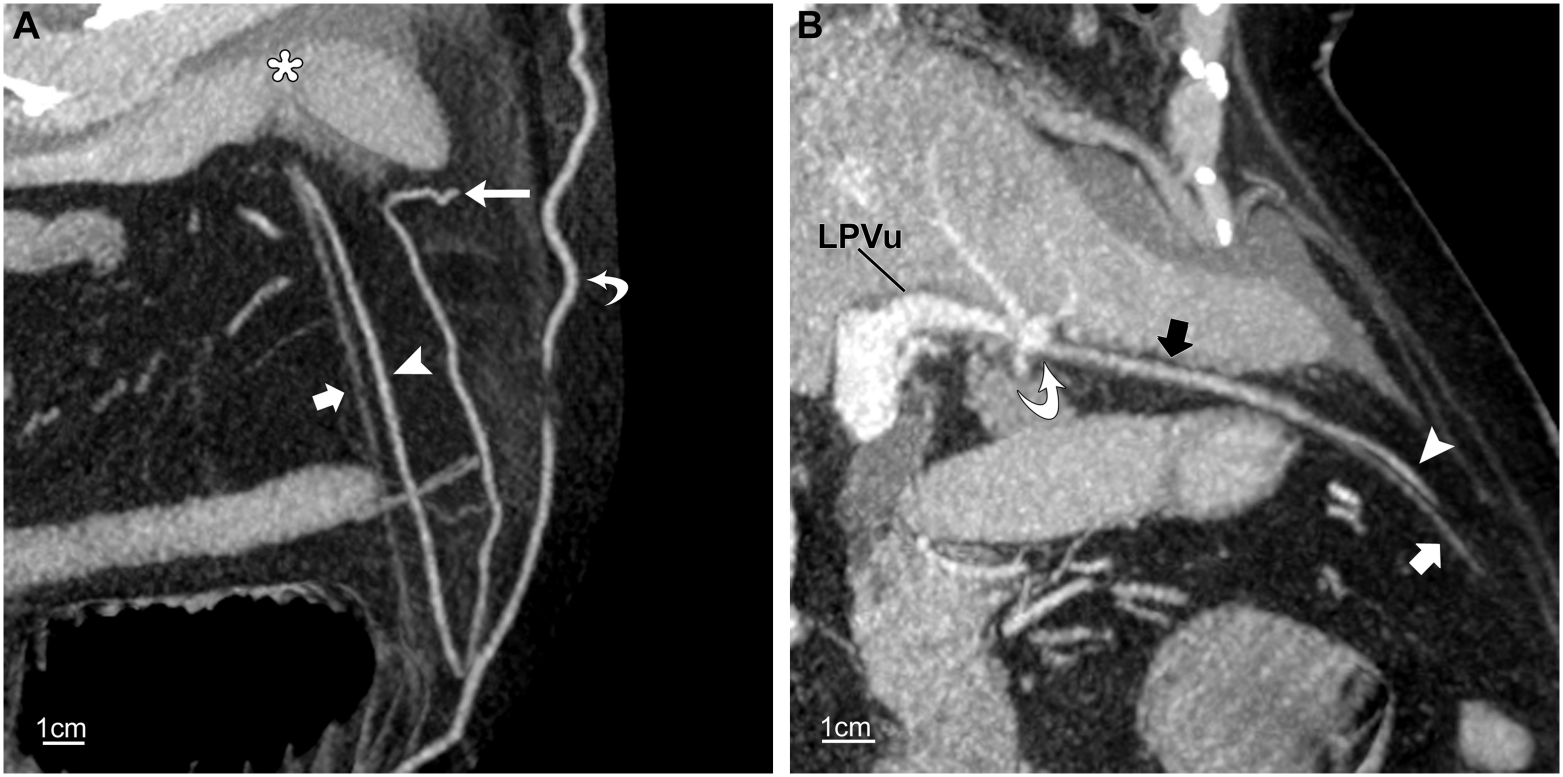
Inferior paraumbilical vein. Routine contrast-enhanced CT of abdomen in a 69-year-old woman with SVC obstruction. (A, B) Oblique sagittal 7-mm maximum-intensity-projections in different planes show an inferior paraumbilical vein (arrowheads), fed by an ensiform vessel (long arrow, A), draining into the umbilical vein (short arrows) just before the liver (asterisk, A). The opacified umbilical vein (black arrow, B) enters the recessus umbilicalis (curved arrow, B) of the left portal vein (LPVu). The umbilical vein below the union (short white arrows) is smaller and not opacified. Also shown is a collateral subcutaneous vein (curved arrow, A).

**Fig 5:**
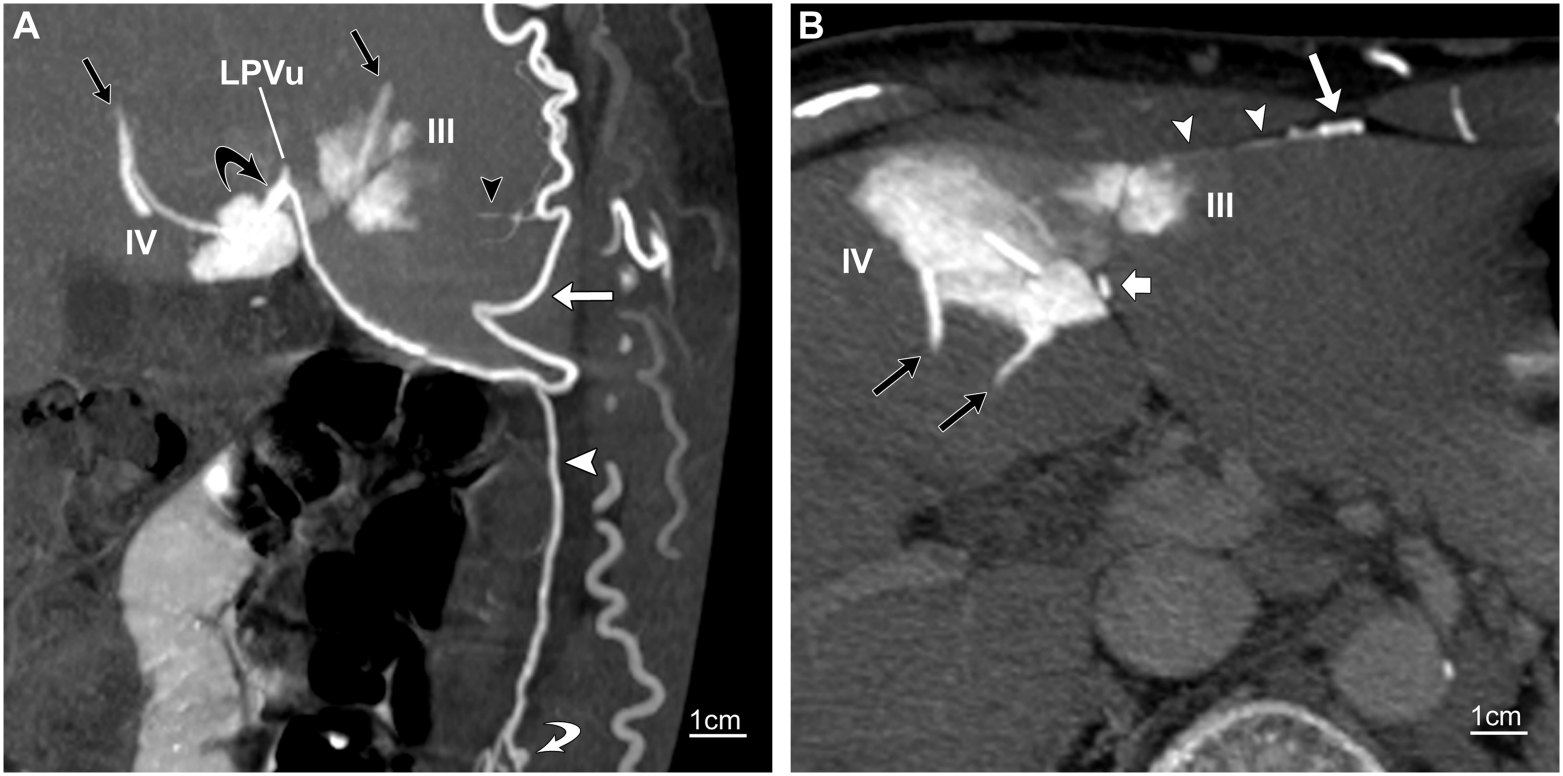
Inferior and superior paraumbilical veins. CT venogram of chest and abdomen in a 53-year-old man with SVC obstruction. (A) Oblique sagittal 10-mm maximum-intensity-projection (MIP) through the umbilical fissure shows an inferior paraumbilical channel (white arrowhead) ascending from deep epigastric veins (curved white arrow). It receives an ensiform vein (straight white arrow) and terminates in the left portal vein (LPVu). Note antegrade filling of a second-order portal branch (curved black arrow) and its territory in segment IV. Also shown are tiny superior paraumbilical veins destined for segment III, which form a tiny venous arch (black arrowhead) and are supplied by the ensiform vein. Straight black arrows point to hepatic veins. (B) Slightly oblique transverse 7-mm MIP shows the collateral channel (short white arrow) in the umbilical fissure and the opacification of segment IV. The tiny superior veins (arrowheads) opacify segment III. The long white arrow points to the ensiform vein; black arrows indicate hepatic veins.

In eight (35%) out of the 23 patients with inferior veins, an inferior vein drained into the umbilical vein (Figs 4 and 5; Parts D—G in S1 Fig). In four of them, this vessel accompanied the main vessel described above, which drained into portal veins at the liver margin. In four patients, the vein could be traced to deep epigastric or subcutaneous veins, and except in one case, also received tributaries continuous with ensiform veins. In the other four cases, the vessel could only be traced to ensiform veins. The inferior vein was opacified by the ensiform veins in seven cases (7/8, 88%), and in two them, it also received collateral flow from deep epigastric veins. In one (1/8, 13%) case, it was opacified only by subcutaneous veins (Table 2).

The inferior vein ascended with the unopacified umbilical vein before entering it near the free margin of the liver in seven cases (7/8, 88%) (Fig 4). In the remaining case (Fig 5A), the unopacified part of the umbilical vein was not seen and might have been obscured by the adherent collateral parambilical vein.

Contrast medium introduced into the LPVu by the umbilical vein was distributed to segments IV and III (Table 3; Fig 5). In two cases, the paraumbilical and umbilical veins were insufficiently filled to opacify the liver.

### Superior veins

**Fig 6:**
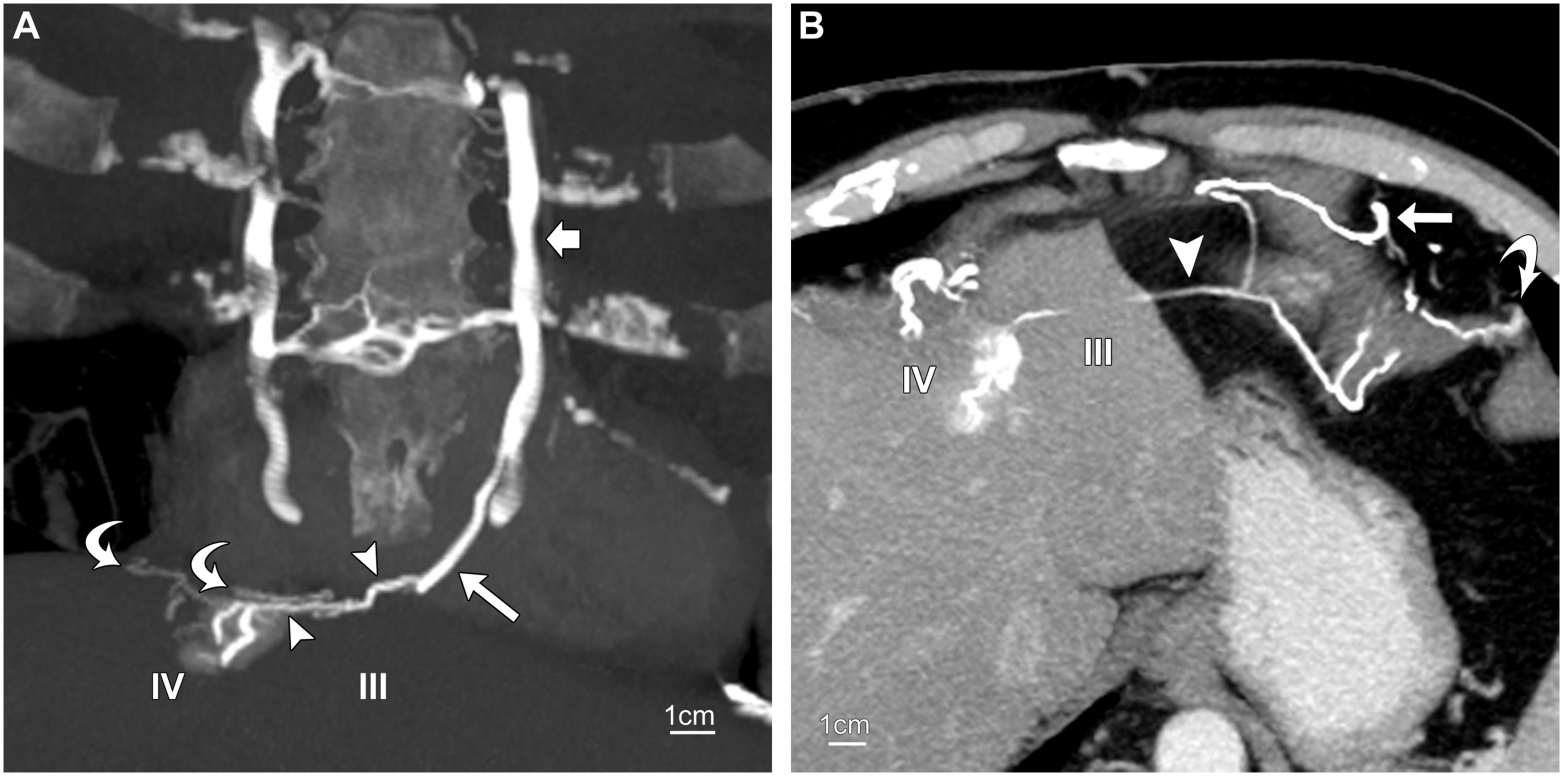
Superior paraumbilical veins. Routine contrast-enhanced CT of chest in a 56-year-old woman with anatomic compression of the left brachiocephalic vein. Slightly oblique coronal 15-mm maximum-intensity-projection (MIP) shows a superior vein (arrowheads) emptying into segment IV at the top of the liver. It communicates with a medial mediastinal branch (long arrow) of the left internal thoracic vein (short arrow) that traverses Morgagni’s foramen and supplies right diaphragmatic veins (curved arrows) as well. (B) Delayed phase contrast-enhanced CT of chest and abdomen in a 57-year-old man with SVC obstruction. Slightly oblique transverse 7-mm MIP shows a superior vein (arrowhead) along the dome of the liver terminating in segment IV. It receives collateral flow from diaphragmatic branches of a left anterior mediastinal vein (straight arrow) in the cardiophrenic fat pad and the left pericardiacophrenic vein (curved arrow). The mediastinal vein descended in the anterior mediastinum from the left brachiocephalic vein.

Superior veins were observed in 17 patients (17/28, 61%) (Figs 5 and 6; Parts F—H in S1 Fig) and were smaller than the inferior veins. In 13 of them (13/17, 76%), a single vessel descended a variable distance along the liver to terminate at its upper convex surface, or it entered the dome of the liver. A medial mediastinal branch of one or both internal thoracic veins supplied almost all of these veins and also gave divisions to the sternal and costal fibers of the diaphragm as it traversed the muscle or Morgagni’s foramen (Fig 6A). Other sources of collateral flow to these superior veins were small diaphragmatic veins opacified by left pericardiacophrenic and anterior mediastinal veins (Fig 6B) (Table 2). In the remaining patients (4/17, 24%), superior veins situated caudally were supplied by ensiform veins and drained into the lower anterior surface of the liver above the umbilical notch (Fig 5).

The superior veins produced a focal area of contrast-enhancement with variable opacification of terminal portal branches in segments IV (14/17, 82%) and III (1/17, 6%) (Table 3).

### Ensiform veins

Collateral ensiform veins were observed in 22 patients. They measured 0.5–4 mm in diameter and often descended in pairs. In the majority of the cases (19/ 22; 86%), they were opacified by a transverse vein that bridged the right and left internal thoracic veins across the xiphoid process (Table 4). They also received collateral flow from the right superior epigastric (6/22; 27%), left superior epigastric (3/22, 14%), subcutaneous (3/22, 14%), right diaphragmatic 1/22, 5%) and anterior mediastinal (1/22, 5%) veins. In three cases (3/22, 14%), the ensiform veins also drained into adjacent left diaphragmatic veins (Fig 4B), tributaries of the left inferior phrenic vein.

**Table 4:** Systemic connections of collateral ensiform veins (*N = 22 patients*).

*Systemic veins*	*No*. *(%) of Patients*
Internal thoracic veins^a^	19 (86)
Right superior epigastric veins	6 (27)
Diaphragmatic veins	4 (18)
Subcutaneous veins	3 (14)
Left superior epigastric veins	3 (14)
Mediastinal veins	1 (5)

^a^via a transverse branch at the xiphoid process

## Discussion

**Fig 7:**
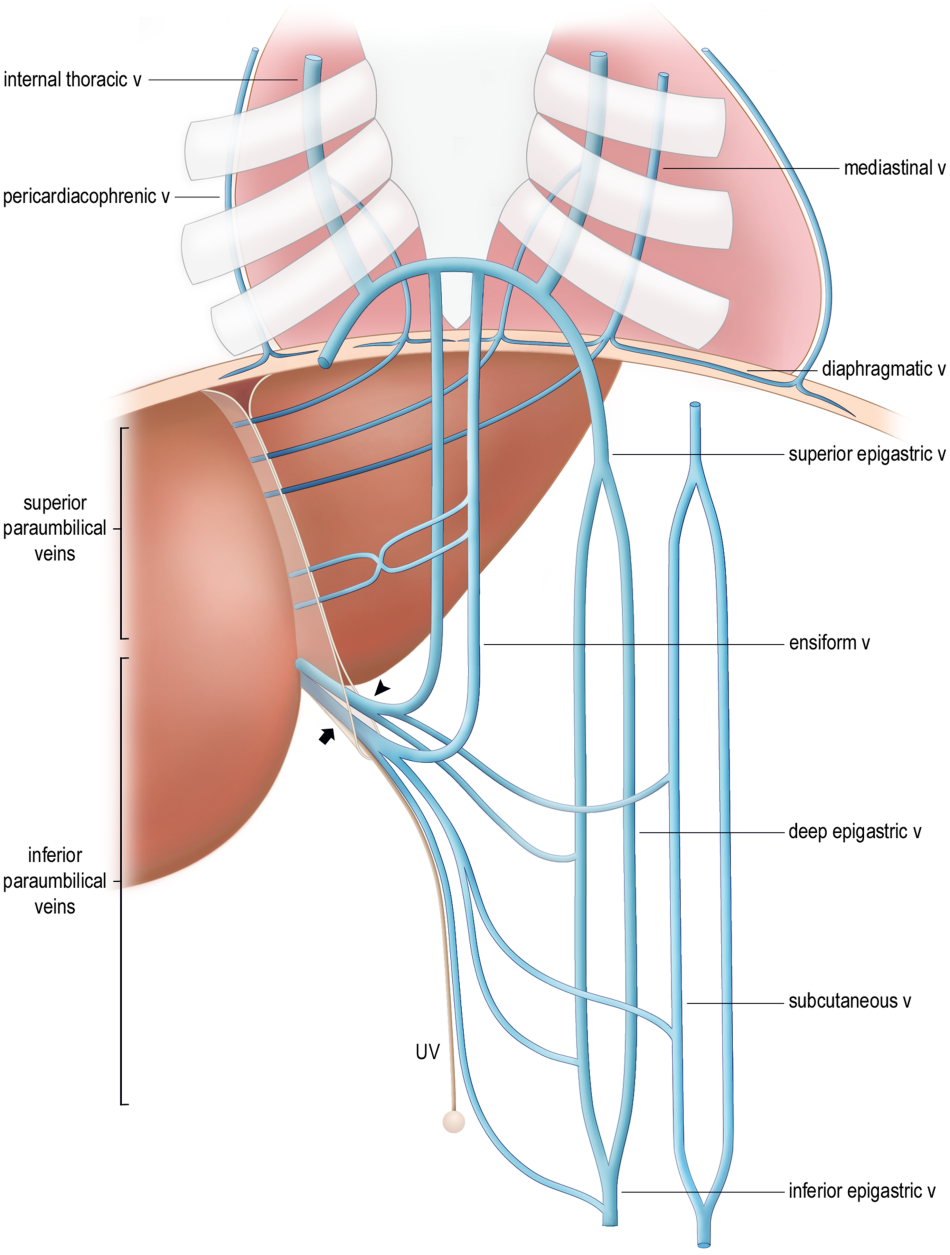
Visual model of the arrangement and systemic anastomoses of collateral paraumbilical veins. The potential collateral pathways between the systemic and paraumbilical veins are shown. Only the left epigastric and subcutaneous vessels are included. An inferior vessel (arrowhead) terminates at the umbilical notch and receives tributaries continuous with ensiform veins, and deep epigastric and subcutaneous veins, which cross the rectus sheath or linea alba (not shown). The ensiform veins descend from a transverse branch of the internal thoracic veins at the xiphoid process. The patent part (arrow) of the umbilical vein (UV) also receives an inferior vein with its tributaries. The obliterated umbilical vein joins the umbilicus. The superior veins communicate with diaphragmatic branches of the internal thoracic, anterior mediastinal and pericardiacophrenic veins. (Visual model created by MUHC Patient Education Office).

In the present investigation, we studied collateral paraumbilical veins in patients with superior venous obstruction using CT in an attempt to elaborate on their connections with the systemic vessels, particularly the ensiform veins. It is recalled that a bolus of contrast material injected in a patient follows the path of least resistance, unlike the contrast material injected in a post-mortem study, which flows in all directions. Furthermore, the capacity of the paraumbilical veins as collateral channels in SVC obstruction depends on many factors including their size and number, the availability of other venous collaterals, and both the pressure gradient along them and the transmural pressure difference [21]. Despite these limitations, we were able to identify superior and inferior veins and document their systemic and portal connections. Fig 7 is a visual model of the potential collateral pathways between the systemic vessels and the different paraumbilical veins based on our observations.

Inferior paraumbilical vessels were observed in 82% (23/28) of our cases. In 19 patients (19/28, 68%), a main vessel communicated directly with the portal veins at the umbilical notch similar to the main channel described by Sappey [3, 4], and Martin & Tudor [1]. The inferior vein that drained into the umbilical vein in eight patients (8/28, 29%) could be a Sappey’s inferior vein or a Burow’s vein.

A little over half of the total number of inferior paraumbilical veins (14/27, 52%) could be traced to deep epigastric or subcutaneous veins, and only eight vessels (8/27, 30%) received collateral flow from these veins. But almost all (26/27, 96%) were connected to the ensiform veins, which were a source of collateral flow to 25 (93%) of them (Table 2). Although observed by Braune [5] and alluded to by Ikuburo et al. [8], the ensiform vessels were not mentioned by other investigators [1, 3, 4, 17], possibly because of insufficient abdominal wall in many of their specimens.

In a study on the properitoneal fat pad in prenatal and neonatal specimens, Merklin [10] observed that as the fetus develops, the fat pad comes to lie in the base of the falciform ligament, and tiny arteries and veins arise from the inferior surface of the liver that pass to the dorsal surface of the fat pad. Although Merklin did not name these hepatic vessels and was unable to demonstrate anastomoses with the ensiform veins, we could assume that the venous channels were paraumbilical veins. Our observations, in keeping with those of Braune [5], show that anastomoses between Sappey’s inferior veins and the ensiform vessels exist. We found anastomoses with both the main channel that emptied into the liver directly and with the veins that drained into the umbilical vein. Sappey’s inferior veins and probably Burow’s veins, then, join the portal system to the venous circulations of the anterior trunk, diaphragm and mediastinum, either directly or via the ensiform vessels (Table 4).

The collateral subcutaneous vessels observed in our cases comprised the superficial epigastric and thoracoepigastric veins, and midline veins called the venae mediana xyphoidea tegumentosa [5, 22]. In some of our cases, the latter formed a superficial pathway between the internal thoracic veins at the xiphoid process and the inferior paraumbilical veins across the linea alba (Fig 3C).

Superior collateral vessels consistent with Sappey’s superior veins [1, 3, 4, 8] were observed in 61% (17/28) of our patients. Sappey [3] noted that these tiny vessels did not always exist, but were of interest because of the connection they establish between the portal circulation and the veins of the diaphragm. More recently, Ibukuro et al. [8] showed that Sappey’s superior veins are also connected to the internal thoracic veins through a medial branch that originates in the lower mediastinum. In the majority of our patients (13/17, 76%), the superior veins communicated with diaphragmatic branches of the internal thoracic, pericardiacophrenic and anterior mediastinal veins (Table 2). In a small number of cases (4/17, 24%), more caudal superior veins were supplied by ensiform veins instead of diaphragmatic veins and drained into the lower anterior surface of the liver [4].

In the normal subject, the paraumbilical veins are small vessels with valves that direct flow toward the liver [3, 5]. The systemic veins communicate with the paraumbilical veins as tributaries with valves at the point of entry or by direct continuity [1]. The few valves present in the ensiform veins direct flow upwards [5]. Martin and Tudor concluded that Burow’s veins, the umbilical vein, and the inferior and superior veins of Sappey form an ascending series, each part linking the portal system with the veins of the anterior abdominal wall at successively higher levels. From our observations, the paraumbilical veins also communicate with the ensiform veins in an ascending order, which form a separate longitudinal pathway that runs parallel to the deep epigastric and subcutaneous vessels. These parallel groups of veins anastamose freely and accommodate an ebb and flow of blood influenced by physiological fluctuations in portal and systemic venous pressures.

Table 2 shows that the most common sources of collateral blood flow to the paraumbilical veins in patients with superior venous obstruction are the ensiform and diaphragmatic branches of the internal thoracic veins. The paraumbilical veins can also afford a collateral pathway to the heart in patients with portal hypertension [3, 4, 6 – 8, 13, 14, 17]. Any of the paraumbilical veins can dilate and shunt portal blood to the systemic veins; however, their frequencies may be different from those observed in this study.

In SVC obstruction and in portal hypertension, collateral flow through the paraumbilical veins might be more desirable than through other visceral venous channels, namely, the bronchopulmonary and gastroesophageal veins [23]. These vessels, unlike the paraumbilical veins, can form submucosal varices or right-to-left shunts, or cause paradoxical embolism. However, a paraumbilical porto-caval shunt in portal hypertension does not prevent the development of gastroesophageal varices and increases the risk of hepatic encephalopathy [24, 25].

Sharp changes in intra-abdominal or intra-thoracic pressure during physical activity, coughing, or Valsalva and Müller maneuvers could affect the pressure gradients along the paraumbilical veins and facilitate the passage of organisms or tumor cells into them at either end. Once inside the paraumbilical veins, organisms and tumor cells have access to an extensive porto-systemic venous network. The paraumbilical veins have been implicated in the spread of hepatobiliary malignancy or infection to the falciform ligament, anterior abdominal wall and diaphragm [26, 27]. They may also play a role in dissemination of hepatobiliary malignancy to unusual distant sites, bypassing the lung [28, 29]. The venous spread of disease to the liver could conceivably occur in a reverse fashion, from the anterior thoracoabdominal wall, diaphragm or mediastinum.

It is important for radiologists to recognize the paraumbilical and ensiform veins in both normal and expanded states. Naturally minute and elusive vessels, paraumbilical veins can sometimes be seen joining the liver at focal low-density spots around the falciform ligament in portal-phase CT scans, supporting the assumption of pseudolesions [30]. Dilated paraumbilical and ensiform veins indicate caval or portal obstruction, and might serve as alternate routes for catheterization of the portal circulation in patients requiring embolization of bleeding varices or of hepatic tumors [31, 32].

## Conclusion

The paraumbilical veins communicate with ensiform, deep epigastric, subcutaneous and diaphragmatic veins. They join the liver to the properitoneal fat pad, anterior trunk, diaphragm and mediastinum. In SVC obstruction, the most common sources of collateral blood flow to the paraumbilical veins are the ensiform and diaphragmatic branches of the internal thoracic veins.

## Supporting information

S1 Fig Schematic diagrams showing the arrangement and frequencies of the collateral paraumbilical veins. (Parts A—E) Inferior veins (single black arrowheads) terminating at the umbilical notch of the liver were observed in 19/28 patients. They received collateral flow (arrows) from ensiform veins, or deep epigastric or subcutaneous veins that pierced the rectus sheath or linea alba. (Parts D—G) In 8/28 patients, separate inferior veins (double black arrowheads) drained into the upper part of the umbilical vein (UV) and were also supplied by collateral ensiform, deep epigastric or subcutaneous veins. (Parts F—H) Superior veins (white arrowheads) were observed in 17/28 patients and emptied into the anterior surface of the liver above the umbilical notch. They received collateral flow from diaphragmatic or ensiform veins. Eight of the 13 patients with diaphragmatic sources (Part H) also had collateral inferior veins (not shown).
(TIF)
