# Supplementary figures and images for: CT of the paraumbilical and ensiform veins in patients with superior vena cava or left brachiocephalic vein obstruction
Source: PLoS One. 2018 Apr 26;13(4):e0196093. doi: 10.1371/journal.pone.0196093 (PMC5919579; doi:10.1371/journal.pone.0196093)

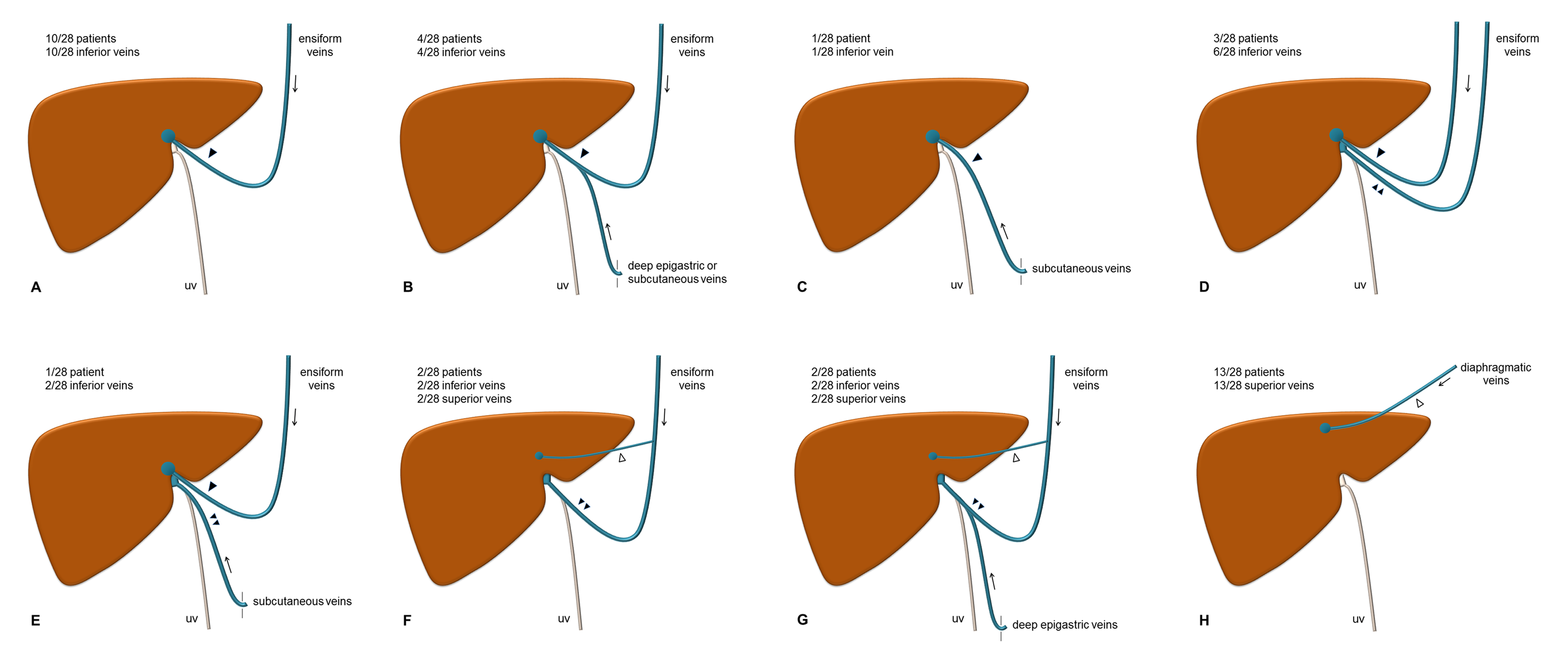

Supplement: S1 Fig — (Parts A—E) Inferior veins (single black arrowheads) terminating at the umbilical notch of the liver were observed in 19/28 patients. They received collateral flow (arrows) from ensiform veins, or deep epigastric or subcutaneous veins that pierced the rectus sheath or linea alba. (Parts D—G) In 8/28 patients, separate inferior veins (double black arrowheads) drained into the upper part of the umbilical vein (UV) and were also supplied by collateral ensiform, deep epigastric or subcutaneous veins. (Parts F—H) Superior veins (white arrowheads) were observed in 17/28 patients and emptied into the anterior surface of the liver above the umbilical notch. They received collateral flow from diaphragmatic or ensiform veins. Eight of the 13 patients with diaphragmatic sources (Part H) also had collateral inferior veins (not shown). (TIF) [file pone.0196093.s001.tif]
